# Pulmonary hypertension and the role of MRI flow assessment: a systematic review

**DOI:** 10.1093/bjr/tqaf182

**Published:** 2025-07-25

**Authors:** Khalid S Alghamdi, Ahmed Maiter, Georgia A Hyde, Turki Alnasser, Michael Sharkey, Mahan Salehi, Pankaj Garg, Jim M Wild, David Kiely, Andrew J Swift, Samer Alabed

**Affiliations:** School of Medicine & Population Health, The University of Sheffield, Sheffield S10 2RX, United Kingdom; Department of Radiological Sciences, College of Applied Medical Science, Imam Abdulrahman Bin Faisal University, Dammam 34212, Saudi Arabia; School of Medicine & Population Health, The University of Sheffield, Sheffield S10 2RX, United Kingdom; Department of Clinical Radiology, Sheffield Teaching Hospitals, Sheffield S10 2JF, United Kingdom; NIHR Sheffield Biomedical Research Centre, University of Sheffield, Sheffield S10 2JF, United Kingdom; Department of Clinical Radiology, Sheffield Teaching Hospitals, Sheffield S10 2JF, United Kingdom; School of Medicine & Population Health, The University of Sheffield, Sheffield S10 2RX, United Kingdom; School of Medicine & Population Health, The University of Sheffield, Sheffield S10 2RX, United Kingdom; School of Medicine & Population Health, The University of Sheffield, Sheffield S10 2RX, United Kingdom; Department of Clinical Radiology, Sheffield Teaching Hospitals, Sheffield S10 2JF, United Kingdom; Norwich Medical School, Faculty of Medicine and Health Sciences, University of East Anglia, Norwich NR4 7TJ, United Kingdom; School of Medicine & Population Health, The University of Sheffield, Sheffield S10 2RX, United Kingdom; Department of Clinical Radiology, Sheffield Teaching Hospitals, Sheffield S10 2JF, United Kingdom; NIHR Sheffield Biomedical Research Centre, University of Sheffield, Sheffield S10 2JF, United Kingdom; Insigneo Institute, Faculty of Engineering, The University of Sheffield, Sheffield S1 3JD, United Kingdom; School of Medicine & Population Health, The University of Sheffield, Sheffield S10 2RX, United Kingdom; NIHR Sheffield Biomedical Research Centre, University of Sheffield, Sheffield S10 2JF, United Kingdom; Insigneo Institute, Faculty of Engineering, The University of Sheffield, Sheffield S1 3JD, United Kingdom; Sheffield Pulmonary Vascular Disease Unit, Sheffield Teaching Hospitals NHS Trust, Sheffield S10 2JF, United Kingdom; School of Medicine & Population Health, The University of Sheffield, Sheffield S10 2RX, United Kingdom; Department of Clinical Radiology, Sheffield Teaching Hospitals, Sheffield S10 2JF, United Kingdom; NIHR Sheffield Biomedical Research Centre, University of Sheffield, Sheffield S10 2JF, United Kingdom; Insigneo Institute, Faculty of Engineering, The University of Sheffield, Sheffield S1 3JD, United Kingdom; School of Medicine & Population Health, The University of Sheffield, Sheffield S10 2RX, United Kingdom; Department of Clinical Radiology, Sheffield Teaching Hospitals, Sheffield S10 2JF, United Kingdom; NIHR Sheffield Biomedical Research Centre, University of Sheffield, Sheffield S10 2JF, United Kingdom; Insigneo Institute, Faculty of Engineering, The University of Sheffield, Sheffield S1 3JD, United Kingdom

**Keywords:** pulmonary hypertension, cardiac MRI flow, right heart catheterization, diagnostic accuracy

## Abstract

**Objectives:**

Cardiac magnetic resonance imaging (CMR) plays an increasingly important role in non-invasive assessment of pulmonary hypertension (PH). This systematic review aimed to assess the utility, accuracy, and clinical applications of CMR flow techniques in evaluating pulmonary arterial blood flow in patients with suspected or confirmed PH.

**Methods:**

MEDLINE and EMBASE databases were searched on December 10, 2024, utilizing the following key terms: “cardiac MRI,” “flow,” and “pulmonary hypertension.” Eligible studies were screened, and data extraction included study design, cohort characteristics, CMR flow techniques and outcomes. Risk of bias was assessed using the Newcastle-Ottawa scale.

**Results:**

Thirty-eight studies (mean sample size: 30 [20-57]) published between 2012 and 2024 were included. These utilized 2D flow (19 studies), 4D flow (15 studies), black blood imaging (1 study) and combined flow techniques (3 studies). Vortex duration derived by 4D flow demonstrated the strongest correlation (*r = *0.96) with mean pulmonary artery pressure and the highest diagnostic accuracy in identifying PH patients (area under the curve, 0.99). Risk of bias rated 14 studies as good/very good and 13 as unsatisfactory, with none justifying their sample size selection.

**Conclusion:**

CMR flow parameters correlate strongly with right heart catheterization measurements and demonstrate high diagnostic accuracy in identifying patients with PH, with 4D flow potentially adding greater value. This systematic review reinforces the potential benefit of CMR flow techniques in the investigation, prognostication, and monitoring of PH patients.

**Advances in knowledge:**

This systematic review is the first to evaluate the role of CMR flow techniques in PH and should inform guidelines on flow assessment in PH.

## Introduction

Pulmonary hypertension (PH) is characterized by elevated pressures and abnormal flow within the pulmonary arterial system, and can arise due to a range of causes. Prompt diagnosis and treatment are important, as PH eventually leads to right ventricular failure which carries considerable morbidity and mortality.^1^ Right heart catheterization (RHC) is the current gold standard investigation for diagnosis of PH, with measurement of a mean pulmonary arterial pressure (mPAP) >20 mm Hg considered diagnostic. However, RHC is invasive, carries the risk of various procedural complications, and is only performed in specialized centres.^2^^,^^3^ Although non-invasive imaging methods are yet to replace RHC for diagnosis of PH, they nonetheless play an important role in the investigation of the disease.

Cardiac magnetic resonance imaging (CMR) is increasingly used for evaluation of PH, and its role is recognized in the 2022 ESC/ERS guidelines.^2^ CMR not only allows accurate non-invasive assessment of cardiac anatomy, but also yields quantitative metrics about cardiac function which have value for patient monitoring and prognostication.^4^ Specialized CMR techniques can also visualize and quantify blood flow through the right ventricle and pulmonary arteries, potentially aiding understanding of the disordered haemodynamics present in PH.^5^^,^^6^

CMR techniques including 2D flow, 4D flow, and black blood imaging play distinct roles in PH assessment, each with specific advantages and trade-offs. Two-dimensional flow imaging is a widely adopted technique due to its efficiency and simple analysis, facilitating the measurement of blood flow volume and velocity. However, 2D flow is limited by the need for predefined single imaging plane and its inability to capture complex flow pattern.^7^ In contrast, 4D flow offers a more detailed assessment by capturing a 3D visualization of the blood flow throughout the cardiac cycle, allowing a better understanding of the pulmonary artery haemodynamics.^8^ In healthy subjects, blood travels smoothly through the pulmonary arteries in regular paths. On the other hand, in the presence of increased mPAP, irregular blood flow patterns, known as vortex, can be observed within the pulmonary artery using 4D flow imaging.^6^ Despite its advantages, 4D flow requires a longer scan time and higher postprocessing demands compared to 2D flow.

Black blood imaging, another CMR technique, is designed to suppress the signal from flowing blood, resulting in dark coloured blood images.^9^ Vessels with fast flowing blood, such as the aorta, achieve excellent signal suppression. On the contrary, it is less effective in regions with slow or turbulent blood flow, such as the pulmonary arteries of patients with PH.^10^ Black blood MRI mainly offers detailed vessel wall imaging and visualization of regional slow blood flow but does not provide quantitative data of flow velocity and direction.^9^

Previous reviews have evaluated the role of 4D flow CMR in the assessment of mitral regurgitation^11^ and assessment of right ventricular diastolic function.^12^ A recent systematic review focused on evaluating the accuracy of CMR 4D aortic flow measurements compared to 2D flow and echocardiography.^8^ This systematic review aims to assess the utility, accuracy and clinical application of CMR flow techniques in assessing pulmonary arterial blood flow in the context of PH.

## Methods

The study protocol was prospectively registered with The International Prospective Register of Systematic Reviews (PROSPERO; CRD42024611433) and the systematic review was conducted according to the Preferred Reporting Items for Systematic Reviews and Meta-Analyses (PRISMA) guidelines.^13^ All figures were generated using GraphPad Prism (version 10.4.1).

### Search strategy and selection

A search was performed in MEDLINE and EMBASE databases on December 10, 2024, using the following key terms and their variations: “cardiac MRI”, “flow,” and “pulmonary hypertension.” The full search strategy is provided as supplementary material (Table S1). Peer-reviewed original research publications until December 2024 were eligible for inclusion if they included adults with suspected or confirmed PH and assessed pulmonary artery flow using CMR. The following study types were excluded: non-human or in silico studies, conference abstracts, preprints, non-English language publications, and studies in which subjects were <18 years of age. Search results were screened for eligibility by 2 authors (K.S.A. and S.A.) independently by reviewing titles and abstracts using Rayyan Systematic Review Screening software,^14^ then followed by full-text assessment. One author (K.S.A.) extracted data from the included studies using a standardized spreadsheet. The data were extracted from full-texts including the country of origin, study design, study population characteristics, flow assessment method, RHC and MRI interval time, and outcome data. The origin of the study was determined based on the first author’s country.

### Risk of bias assessment

The risk of bias was assessed in all included studies as a part of the data extraction process using the Newcastle-Ottawa scale (NOS).^15^ The criteria included 3 main domains: selection, comparability, and outcome. The studies were scored from 0 to 10 based on the NOS into 4 different categories: unsatisfactory, satisfactory, good, and very good. Risk of bias assessment was performed by K.S.A. and reviewed by S.A. Any disagreements were resolved by consensus agreement.

## Results

**Figure 1.**
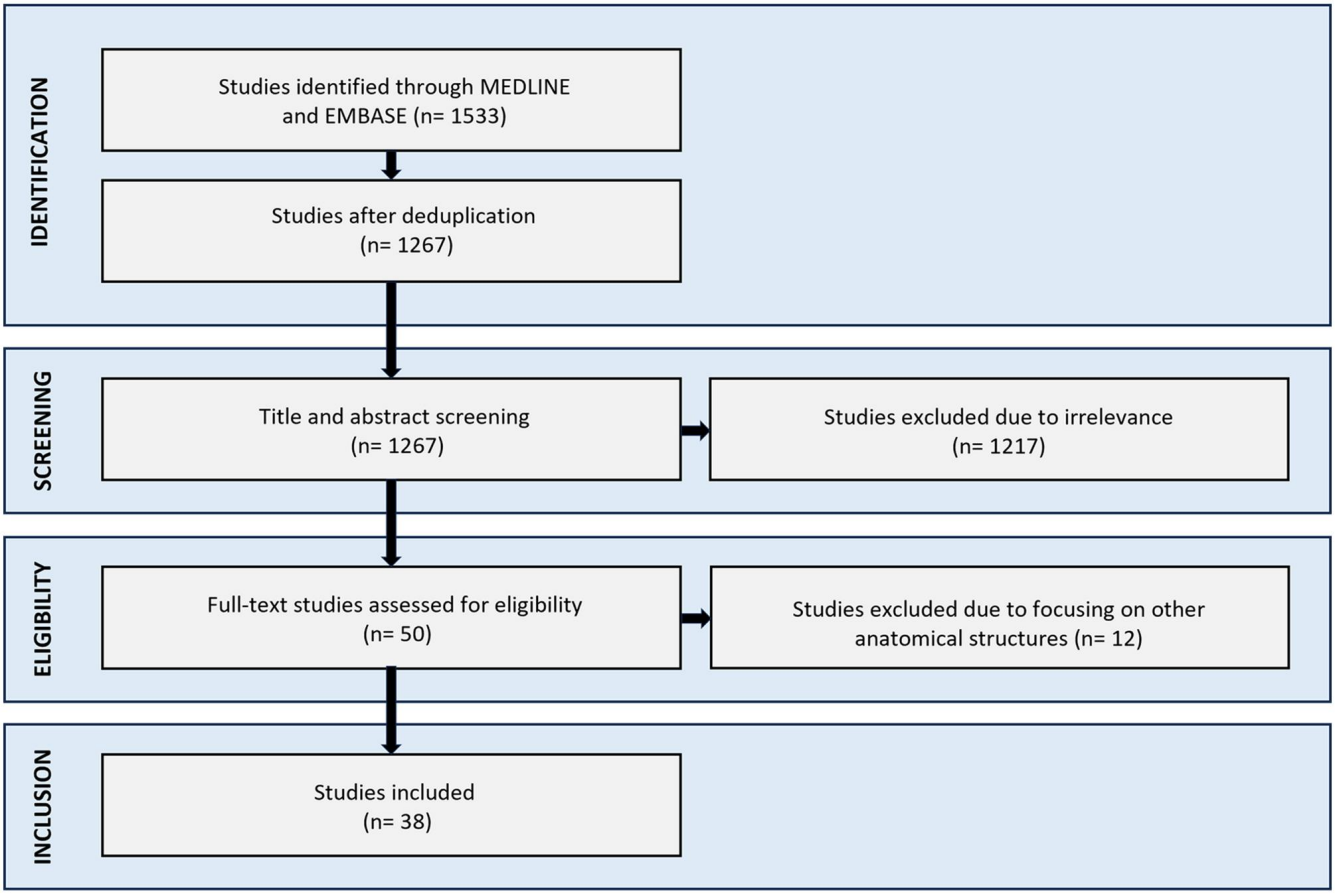
PRISMA diagram showing the studies selection and inclusion process.

**Figure 2.**
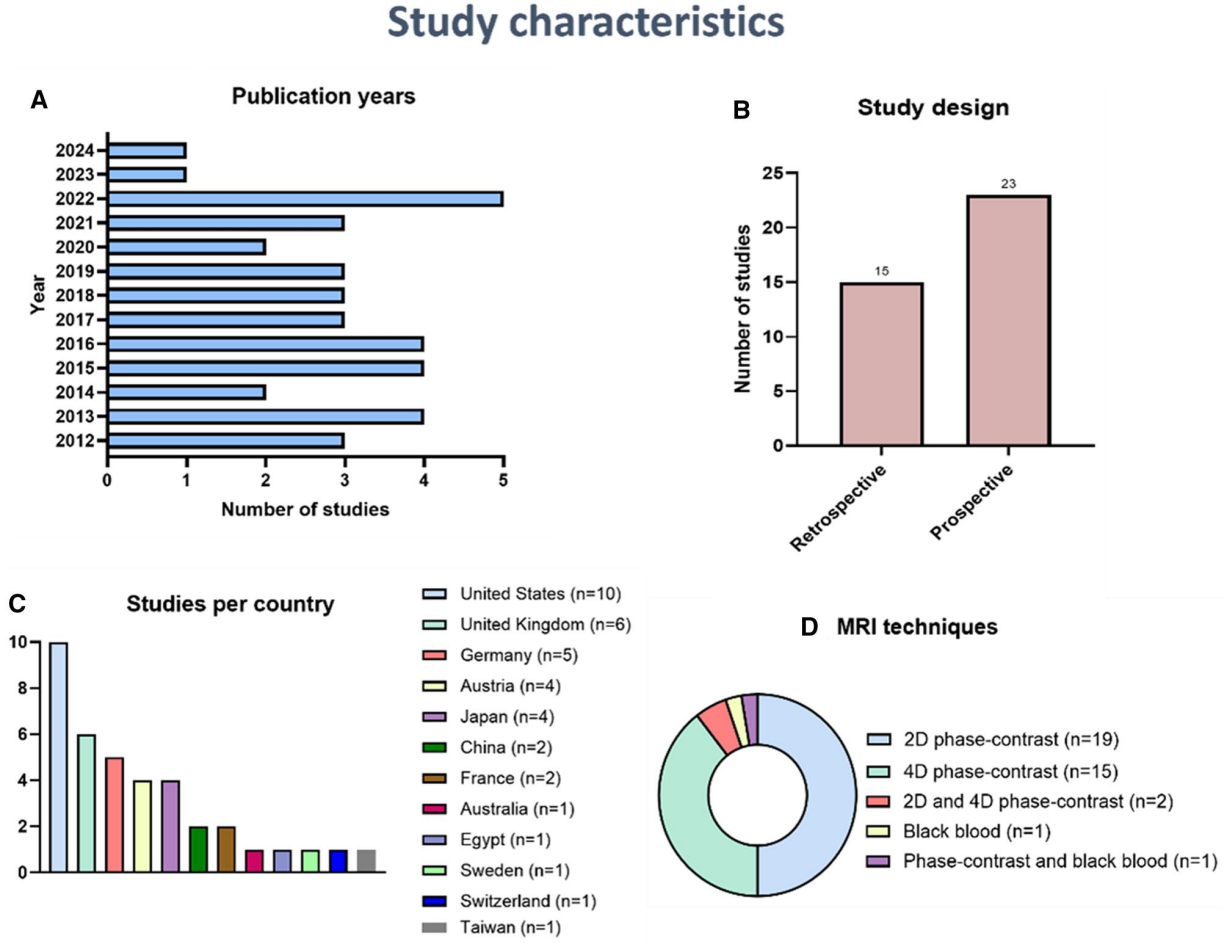
Descriptive details of the included studies (A) shows the years of publications (B) demonstrates the study design (C) shows the origin of the studies (D) summarizes the used MRI techniques.

Thirty-eight studies were included in this systematic review (Figure 1). Descriptive information of the included studies is summarized in Figure 2. The studies were published between 2012 and 2024 originating from 12 different countries (Figure 2C). Twenty-three (61%) of the studies were prospective, while 15 (39%) were retrospective (Figure 2B). Nineteen (50%) studies used 2D phase-contrast technique to assess the pulmonary artery blood flow, followed by 4D phase-contrast in 15 (39%) studies, and 2 (5%) studies used both techniques. One (3%) study used the black blood imaging technique to assess the pulmonary artery flow, while another (3%) study used both black blood and phase contrast MRI techniques (Figure 2D).

The mean sample size for all studies was 30 (20-57) participants per study. Nine (24%) studies focused on assessing the flow dynamics in patients with chronic thromboembolic PH (CTEPH), 7 (18%) in pulmonary arterial hypertension (PAH) and the remaining (58%) studies in mixed PH. The mean age of participants was 55 ± 12 years, with a mean mPAP of 32 ± 19 mm Hg, and 60% of the study population were female. The time interval between RHC and CMR ranged from within minutes to 1 year. The characteristics of the included studies are summarized in Table 1.

**Table 1.** Characteristics of the included studies.

Author, year	Country	Study design	Population (female %)	Cohort	Age (years)	mPAP (mm Hg)	RHC-MRI interval time (days)
Baillie et al, 2016^16^	Australia	Prospective	20 (55)	PAH	54 ± 14	46 ± 16	2
Bane et al, 2015^17^	United States	Prospective	14 (86)	PH	55 (32-70)^a^	40 ± 14	1-10
Barker et al, 2015^18^	United States	Prospective	36 (47)	PAH	57 ± 10	45 ± 17	NR
Cerne et al, 2022^19^	United States	Prospective	79 (49)	PAH = 11	52 ± 11	46 ± 10	28
				PH-LHD = 24	63 ± 14	35 ± 9	
				PH-CLD = 9	63 ± 11	30 ± 6	
				CTEPH = 10	52 ± 13	38 ± 11	
				Controls = 25	52 ± 14	NA	
Creuzé et al, 2015^20^	France	Prospective	85 (54)	PH = 65	61 (51-71)^a^	44 (38-53)^a^	NR
				No PH = 20	59 (48-64) ^a^	20 (18-23)^a^	
Czerner et al, 2020^21^	Austria	Retrospective	31(35)	CTEPH	53 (49-70)^a^	46 (38-53)^a^	2
Deux et al, 2022^22^	Switzerland	Retrospective	25 (60)	CTEPH	63 ± 16	33 ± 15	39 ± 82
Dong et al, 2022^23^	United States	Prospective	20 (40)	CTEPH	62 ± 14	44 ± 12	NR
Guo et al, 2014^24^	China	Prospective	20 (35)	CTEPH	58 ± 11	47 ± 9	3
Gupta et al, 2018^25^	United Kingdom	Prospective	20 (70)	PH	55 ± 19	NR	<1
Johns et al, 2019^26^	United Kingdom	Retrospective	603 (61)	PH = 506	52 ± 13	47 ± 13	14
				No PH = 97	56 ± 16	19 ± 3	
Kamada et al, 2022^27^	Japan	Prospective	28 (75)	CTEPH	68 (50-83)^a^	40 ± 1.6	363 ± 29
Kawakubo et al, 2017^28^	Japan	Retrospective	3 (100)	CTEPH	63 ± 11	NR	NR
Kheyfets et al, 2016^29^	United States	Prospective	22 (64)	PH = 17	60 ± 10	37 ± 11	<1
				No PH = 5	54 ± 9	20 ± 3	
Kräuter et al, 2022^30^	Austria	Prospective	32 (78)	PH = 19	64± 15	43 ± 15	6±11
				No PH = 13	60 ± 14	14 ± 2	
Kreitner et al, 2013^31^	Germany	Prospective	19 (42)	CTEPH	51 (23-78)^a^	38 (26-45)^a^	<1
Kroeger et al, 2021^32^	Germany	Prospective	14 (36)	PH	61 ± 16	42 ± 13	1-6
Ley et al, 2013^33^	Germany	Prospective	20 (70)	PAH and CTEPH	47 ± 8	48 ± 19	NR
Li et al, 2016^34^	China	Prospective	30 (77)	Mixed PH	28 ± 10	61 ± 19	7
Lin et al, 2024^35^	United States	Retrospective	73 (52)	PH = 50	59 ± 12	NR	NR
				Controls = 23	52 ± 13		
Lungu et al, 2014^36^	United Kingdom	Retrospective	35 (0)	PH	NR	NR	NR
Nagao et al, 2017^37^	Japan	Prospective	24 (79)	CTEPH	61±11	NR	NR
Pewowaruk et al, 2021^38^	United States	Retrospective	15 (87)	PH = 7	55 ± 16	NR	NR
				Healthy = 8	56 ± 14		
Ramos et al, 2020^39^	Sweden	Prospective	60 (37)	Mixed PH	60 (48-68)^a^	NR	NR
Reiter et al, 2021^40^	Austria	Retrospective	44 (59)	Mixed PH = 27	60 ± 14	45 ± 11	8 ± 13
				No PH = 15	57 ± 11	15 ± 2	
Reiter et al, 2013^41^	Austria	Prospective	50 (68)	PH = 23	59 ± 13	41 ± 11	10 ± 14
				No PH = 27	56 ± 13	16 ± 4	
Rolf et al, 2015^42^	Germany	Retrospective	43 (65)	CTEPH	56 ± 16	47 ± 12	1-11
Romeih et al, 2023^43^	Egypt	Retrospective	30 (93)	PH	32 ± 10	63 ± 15	1
Schäfer et al, 2017^44^	United States	Prospective	45 (62)	PH = 35	61 ± 9	36 ± 11	NA
				Controls = 10	57 ± 9	–	
Sieren et al, 2019^45^	Germany	Retrospective	46 (57)	PAH = 11	62 ± 16	46 ± 16	12 ± 15
				Old healthy = 15	56 ± 11	NR	
				Young healthy = 20	23 ± 2	NR	
Stevens 2012^46^	United States	Retrospective	124 (69)	Mixed PH	52 (16-88)^a^	40 (29-50)^a^	1-7
Swift et al, 2013^47^	United Kingdom	Retrospective	128 (60)	Mixed PH	62.9 ± 13.5	39 ± 14	<1
Swift et al, 2012^48^	United Kingdom	Retrospective	233 (72)	PAH = 85	59 ± 16	46 ± 15	2
				PH = 194	64 ± 15	45 ± 13	
				No PH = 39	62 ± 16	20 ± 5	
Swift et al, 2012^49^	United Kingdom	Retrospective	134 (61)	PH = 115	64 ± 14	45 ± 13	2
				No PH = 19	59 ± 18	19 ± 3	
Terada et al, 2016^50^	Japan	Prospective	17 (18)	PAH = 5	77 (73-88)^a^	33 ± 7	NR
				Non-PAH = 12	74 (57-83)^a^	18 ± 3	
Venner et al, 2018^51^	France	Prospective	56 (61)	PH	61 ± 16	NR	1-3
Wang et al, 2019^52^	Taiwan	Prospective	23 (57)	PAH = 11	43 ± 17	NR	NR
				No PH = 12	38 ± 8		
Zambrano et al, 2018^53^	United States	Prospective	2 (0)	PAH = 1	44	NR	NR
				Healthy = 1	65		

Abbreviations: CTEPH = chronic thromboembolic pulmonary hypertension; MRI = magnetic resonance imaging; NA = not applicable; NR = not reported; PAH = pulmonary arterial hypertension; PH-CLD = pulmonary hypertension due to chronic lung disease; PH-LHD = pulmonary hypertension due to left heart disease; RHC = right heart catheterization.

a Indicates that data are presented as median, data in parentheses are ranges.

### Flow metrics

The main findings are classified based on the CMR flow technique. For detailed results, see Table 2.

**Table 2.** Results of the flow CMR parameters of the pulmonary artery in the included studies.

Correlation with invasive RHC measurements										
MRI technique	Author, year	PH type		Flow parameter			mPAP		PVR	
2D flow	Baillie et al, 2016^16^	PAH		Average velocity at hyperaemia			NA		−0.88	
	Guo et al, 2014^24^	CTEPH		Mean blood flow			−0.38		−0.73	
	Gupta et al, 2018^25^	PH		Velocity transfer function of PA			NA		0.63	
4D flow	Cerne et al, 2022^19^	PH-CLD		Peak velocity			NA		−0.73	
	Kroeger et al, 2021^32^	PH		Peak velocity			0.63		NA	
	Deux et al, 2022^22^	CTEPH		Vortex duration			0.75		0.52	
	Reiter et al, 2013^41^	PH		Vortex duration			0.96		NA	
	Kräuter et al, 2022^30^	PH		Vortex visualization			0.98		NA	
	Terada et al, 2016^50^	PAH		Wall shear stress			−0.64		NA	
Black blood	Swift et al, 2012^48^	Mixed PH		Pulmonary flow artefact			0.65		0.70	

**PH diagnostic accuracy**										
MRI technique	Author, year	PH type			Flow parameter	Sensitivity (%)		Specificity (%)		AUC
2D flow	Swift et al, 2012^49^	Mixed PH			RAC ≤ 15%	84		74		0.87
	Creuzé et al, 2015^20^	CTEPH			RAC ≤ 20%	77		85		0.84
					Vmean < 10 cm/s	66		100		0.89
	Nagao et al, 2017^37^	CTEPH			PA energy < 45 J/kg	78		92		0.91
	Pewowaruk et al, 2021^38^	PH			Pulse wave velocity	NR		NR		0.91
	Wang et al, 2019^52^	PAH			Wall shear stress	70		100		0.85
					Acceleration time	90		91		0.92
	Johns et al, 2019^26^	Mixed PH			Model 1	93		79		0.95
					Model 2	92		59		0.93
4D flow	Sieren et al, 2019^45^	PH			Minimum area ≤ 660 mm^2^	91		93		NR
	Deux et al, 2022^22^	CTEPH			Vortex duration > 8.6%	95		83		0.86
	Reiter et al, 2013^41^	PH			Vortex duration > 15%	100		96		0.99
	Schäfer et al, 2017^44^	PH			Helicity ≤ 75.2 m/s^2^	90		89		0.93
Black blood	Swift et al, 2012^48^	Mixed PH			Pulmonary flow artefact	86		85		NR

Abbreviations: AUC = area under the curve; CTEPH = chronic thromboembolic pulmonary hypertension; ICC = intraclass correlation coefficient; mPAP = mean pulmonary arterial pressure; NA = not applicable; NR = not reported; PAH = pulmonary arterial hypertension; PH-CLD = pulmonary hypertension due to chronic lung disease; PVR = pulmonary vascular resistance; RAC = relative area change; Vmean = mean velocity.

### 2D flow

Two-dimensional flow CMR parameters demonstrated moderate to strong correlations with PVR derived by RHC. Three studies assessed the correlation of 2D flow CMR parameters including mean velocity, mean blood flow, and velocity transfer of PA with RHC-derived PVR. Among these parameters, mean pulmonary artery flow velocity demonstrated inverse correlation (*r = *−0.88) with PVR in patients with PAH (mean mPAP of 46 ± 16 mm Hg).^17^ For PH diagnostic accuracy, 6 studies investigated the role of 2D flow parameters in identifying PH. A mean pulmonary artery flow velocity of <10 cm/s achieved a specificity of 100% in 65 PH patients (mean mPAP of 44 [38-53] mm Hg) and 20 patients with no PH (mean mPAP of 20 [18-32] mm Hg).^20^ Pulmonary artery flow acceleration time yielded a sensitivity of 90% and AUC of 0.92 in identifying PH in 11 patients with PAH (mean mPAP of 43 ± 17 mm Hg) and 10 patients without PH (mean mPAP of 38 ± 8 mm Hg).^52^ 2D flow was also used to assess the response to endarterectomy (PEA) treatment using peak velocity in 3 studies. Rolf et al reported that peak velocity significantly increases post-PEA (73.8 ± 19 cm/s) compared to pre-PEA (60.8 ± 16 cm/s) in 43 patients with CTEPH (*P* = .007),^42^ indicating an improvement in the pulmonary flow.

### 4D flow

Six studies investigated how well 4D flow-derived parameters correlate with mPAP, with 3 studies focusing on PH-related vortex. It has been reported that mPAP estimated via visual detection of vortex (43.3 ± 13.5 mm Hg) strongly correlated (*r = *0.98) with mPAP measured by RHC (42.7 ± 14.6 mm Hg) in 19 patients with PH (13 PAH patients, 4 patients with CTEPH and 2 patients with multifactorial PH).^30^ Moreover, the duration of PH-related vortex was found to have a diagnostic value. Reiter et al reported that vortex duration had a high accuracy for identifying PH with a sensitivity of 100%, specificity of 96% and area under the curve of 0.99 in 23 PH patients with an average mPAP of 41 ± 11 mm Hg, and 27 patients without PH with an average mPAP of 16 ± 4.^41^ Furthermore, visualization of the PH-related vortex in the main and right PA is strongly associated with PVR (*r*^2^ = 0.94).^29^ In comparison with 2D flow, 4D flow demonstrated an excellent agreement in measuring the maximum and minimum pulmonary artery areas, as well as stroke volume (Table 3).^28^^,^^45^

**Table 3.** Agreement of 2D and 4D flow CMR parameters.

Author, year	Cohort	Flow parameters	Agreement
Kawakubo et al, 2017^28^	CTEPH	Stroke volume	*r = *0.91
Sieren et al, 2019^45^	PH	Stroke volume	ICC = 0.82
		Minimum area	ICC = 0.95
		Maximum area	ICC = 0.94

Abbreviations: CMR = cardiac magnetic resonance imaging; CTEPH = chronic thromboembolic pulmonary hypertension; ICC = intraclass correlation coefficient; PH = pulmonary hypertension; r = Pearson correlation coefficient.

### Black blood imaging

A study introduced a visual scoring system of slow blood flow that had a sensitivity of 86% and specificity of 85% in identifying 233 patients with suspected PH. There were good correlations between the pulmonary flow artefact scores and RHC-derived PVR and mPAP (*r = *0.70 and 0.65, respectively).^48^ Moreover, slow blood flow improved the diagnostic accuracy of a regression model in identifying PH, with a sensitivity of 93% and specificity of 79%.^26^

### Risk of bias assessment

In the quality assessment, 4 studies were rated as very good, 10 as good, 11 as satisfactory and 13 as unsatisfactory. None of the studies justified their sample size selection (Figure S1). Detailed results of the risk of bias assessment are attached in the Supplementary Materials (Table S2).

## Discussion

To the best of our knowledge, this is the first systematic review to comprehensively evaluate CMR flow techniques for the assessment of pulmonary artery blood flow in individuals with suspected or confirmed PH. Thirty-eight peer-reviewed journal publications were included. We found that 2D flow parameters demonstrated a moderate to strong correlation with RHC-derived PVR and good value in assessing treatment response. On the other hand, PH-related vortex obtained by 4D flow demonstrated stronger correlation with RHC-measured mPAP and higher diagnostic accuracy in identifying patients with PH compared with 2D flow parameters. Furthermore, our findings have confirmed the potential value of black blood imaging in the assessment of PH. However, the risk of bias assessment highlighted methodological pitfalls in the existing literature, as a considerable number of the studies were evaluated as unsatisfactory.

Haemodynamic measurements derived from RHC were estimated in several studies using CMR flow techniques to evaluate their potential clinical utility. The majority of these studies used 2D flow-derived parameters (eg, minimum area) to estimate RHC-derived mPAP, though with moderate correlation. Other 2D flow-derived parameters, such peak velocity, showed a significant post-treatment increase in patients with CTEPH, indicating improved pulmonary flow.^21^^,^^42^ These findings reflect the value of 2D flow-derived metrics for non-invasive disease monitoring and assessing treatment response in PH patients. However, limitations such as moderate correlations of some parameters and variability in CMR-flow parameters used for mPAP estimation may affect their clinical reliability ability to replace invasive methods.

PVR is an essential haemodynamic parameter for the assessment of PH, and elevation in PVR is associated with disease severity.^54^ Two-dimensional flow parameters such as average velocity and mean blood flow have been reported as an indicator for elevation of PVR in PAH and patients with CTEPH.^16^^,^^24^ Moreover, a study that used black blood imaging technique to assess PA blood flow in a mixed PH cohort showed that the presence of slow blood flow correlated with the elevation of PVR.^48^ Overall, the findings demonstrated a significant association of CMR flow parameters with PVR, with 2D flow-derived parameters achieving the highest correlation, highlighting its potential as a non-invasive tool for assessing PH severity.

Vortex duration was reported as a predictive indicator of elevated mPAP across different PH subgroups, though with variable correlations.^22^^,^^41^ The variability in the correlation may reflect heterogeneity of the patient cohorts in the 2 studies, as different PH subgroups have distinct haemodynamic profiles. The strong correlation between PH-related vortex and mPAP allowed for non-invasive estimation of mPAP based on visual detection of vortices.^30^ However, visual assessment of vortices is limited by a high likelihood of observer bias and interobserver variability. To minimize these issues, the authors introduced an automated method for vortex detection, which strongly correlated with the manual method. The duration of PH-related vortex demonstrated a high diagnostic accuracy in detection of PH.^22^^,^^41^ However, the variability in the cut-off value used for calculating the vortex duration (8.6% vs 15% of the cardiac cycle) can affect the reliability and comparability of the findings, highlighting the need for a standardized method for calculating vortex duration.

In the current 2022 ESC/ERS guidelines, the potential value of CMR is highlighted, but there is no recommendation for it to be used as a diagnostic tool.^2^ Nevertheless, several studies have demonstrated the high diagnostic accuracy of 2D flow-derived metrics such as acceleration time and average velocity of the pulmonary artery blood flow.^20^^,^^52^ In one study, the authors aimed to develop a regression model to predict mPAP using 2D flow and black blood imaging parameters. Two models were developed, with and without slow blood flow derived by black blood imaging. The model that included the black blood parameter achieved higher diagnostic accuracy, highlighting the potential value of black blood imaging in enhancing the diagnosis of PH.^26^

Both 2D and 4D flow CMR techniques showed excellent agreement in measuring the maximum and minimum areas of the pulmonary artery.^45^ Moreover, stroke volume measured by 4D flow did not differ significantly from that measured by 2D flow.^28^ Another study reported that there was no significant difference between stroke volume measured by 2D flow compared to 4D flow. However, the peak velocity of the main pulmonary artery measured by 4D flow was higher than that measured by 2D flow.^55^ The underestimation of peak velocity in 2D flow may result from suboptimal positioning of the imaging plane, leading to misalignment with the flow direction and an inaccurate representation of the peak velocity within the pulmonary artery. In contrast, 4D flow allows retrospective plane adjustment with the aid of 3D anatomical data, allowing perpendicular positioning to the pulmonary artery for accurate measurement.^56^ Overall, 2D flow is widely used for routine assessment of blood flow due to its shorter scan time and straightforward interpretation, making it suitable for follow-up. On the other hand, 4D flow may be favoured in more complex cases (eg, congenital heart disease), where detailed flow analysis is valuable.^57^ Therefore, the choice of CMR flow technique should be based on the clinical question and patient condition.

This systematic review has highlighted the value of CMR flow techniques in the assessment of pulmonary artery flow in PH patients. However, several limitations need to be addressed. The majority of the studies were restricted by small sample size, which may affect the statistical power and validity of their outcomes.^58^ All the 2D flow studies and most of the 4D studies were conducted using single-centre cohorts, likely affecting the generalizability of the findings. Other factors such as long-time intervals between RHC and CMR exams could affect the comparability of the 2 measurements, since PH is a progressive disease. Even though 4D flow achieved higher diagnostic accuracy compared to other CMR techniques, the scan time typically takes 10-20 min, which limits its implementation on patients with breathing difficulties.^19^ Moreover, limited temporal resolution of 4D flow may impact the accuracy of mPAP estimation.^30^ In black blood imaging, the assessment is qualitative, making it prone to subjectivity and less reproducible. Finally, none of the studies justified their sample size selection, as indicated by the risk of bias assessment. There are also limitations in the methodology of this systematic review. The search results and data extraction were conducted by a single observer; while these steps were performed following a structured approach, subjectivity may still occur. Finally, the exclusion of conference papers, case reports, and preprints may introduce publication bias.

Future studies should address the highlighted limitations in this review to strengthen the validity of their findings. Multicentre studies and justified sample sizes are essential for confirming the current findings and enhancing the generalizability. Moreover, same day RHC and CMR exams would minimize any potential bias resulting from the progressive nature of PH, specifically in severe PH patients. Moreover, direct comparison of CMR flow techniques with the non-invasive gold standard method for PH assessment (ie, echocardiography) can give better understanding of their roles in PH diagnosis. Finally, integrating artificial intelligence in analysing pulmonary artery blood flow can help to minimize the subjectivity in identifying slow blood flow and PH-related vortex. The use of automated detection methods has the potential to improve the reproducibility of these flow CMR parameters and limit observer bias. In addition, artificial intelligence can help overcome the drawbacks of 4D flow such as long scan time, which limit its clinical implementation, by accelerating image reconstruction and postprocessing, or by simulating blood flow from morphological MRI images.^59^

## Conclusion

CMR flow parameters correlate strongly with RHC measurements and demonstrate high diagnostic accuracy in identifying patients with PH, with 4D flow techniques potentially adding value over alternative methods. This systematic review is the first to evaluate the role of CMR flow techniques in PH and should inform guidelines on flow assessment in PH.

## Supplementary Material

tqaf182_Supplementary_Data
