# Supplementary material for: Pulmonary hypertension and the role of MRI flow assessment: a systematic review
Source: Br J Radiol. 2025 Jul 25;98(1175):1938–46. doi: 10.1093/bjr/tqaf182 (PMC12659743; doi:10.1093/bjr/tqaf182)
Supplement: tqaf182_Supplementary_Data [file tqaf182_supplementary_data.zip › supplementary materials_21-7.pdf]

Supplementary materials

Supplementary Table 1. EMBASE and MEDLINE used search terms.

**1**	Pulmonary hypertension [MESH]
**2**	Pulmonary hypertension
**3**	Pulmonary vascular disease [MESH]
**4**	Pulmonary vascular disease
**5**	1 or 2 or 3 or 4
**6**	Flow [MESH]
**7**	Flow
**8**	(Pulmon* flow or pulmon* arter* flow)
**9**	6 or 7 or 8
**10**	Nuclear magnetic resonance imaging [MESH] or exp cardiovascular magnetic resonance [MESH] or exp magnetic resonance angiography [MESH]
**11**	(MRI or magnetic resonance)
**12**	Phase contrast
**13**	(4D or four dimension*)
**14**	10 or 11 or 12 or 13
**15**	5 and 9 and 14
**16**	Remove duplicates from 15
**17**	Limit 16 to human

Supplementary Table 2. Results of the risk of bias assessment.

Newcastle-Ottawa Scale adapted for non-randomised studies									
Author	Year	Selection				Comparability	Outcome		Total score
		Representativeness of the sample ★	Sample size ★	Non-respondents ★	Ascertainment of the exposure ★★	Comparability of subjects in different outcome groups ★★	Assessment of outcome ★★	Statistical test ★	
Baillie et al	2017	★		★	★★	★★	★		7
Bane et al.	2015			★	★★	★			4
Barker et al.	2014							★	1
Cerne et al.	2022	★		★	★★	★	★	★	7
Creuzé et al	2015				★★	★	★★		5
Czerner et al.	2020	★		★	★		★	★	5
Deux et al.	2022	★		★	★★		★★	★	7
Dong et al.	2022	★			★★	★★	★★	★	8
Guo et al.	2014			★	★★	★★	★	★	7
Gupta et al	2018	★		★	★★	★★	★	★	8
Johns et al.	2019	★		★	★★	★★	★★	★	9
Kamada et al.	2022				★★	★			3
Kawakubo et al.	2016			★		★★		★	4
Kheyfets et al.	2016	★			★★	★★	★★	★	8
Kräuter et al.	2022	★		★	★★	★★	★★	★	9
Kreitner et al	2013			★	★		★★	★	5
Kroeger et al.	2021			★	★★			★	4
Ley et al.	2013			★		★★	★★		5
Li et al.	2016			★	★★		★★	★	6
Lin et al.	2024	★		★	★		★	★	5
Lungu et al.	2014	★			★★		★★		5
Nagao et al.	2017			★	★★	★		★	5
Pewowaruk et al.	2021	★			★★			★	4
Ramos et al.	2020	★		★			★★	★	5
Reiter et al	2021			★	★★	★★		★	6
Reiter et al.	2013	★		★	★★	★	★★	★	8
Rolf et al.	2014			★	★★	★			4
Romeih et al.	2023			★	★★		★	★	5
Schäfer et al.	2017	★						★	2
Sieren et al.	2019			★	★★			★	4
Stevens et al.	2012	★			★★	★	★★	★	7
Swift et al.	2013	★		★	★★	★★	★★	★	9
Swift et al.	2012	★		★	★★	★★	★★	★	9
Swift et al.	2012	★			★★			★	4
Terada et al.	2016	★			★★			★	4
Venner et al.	2018	★			★★	★★	★	★	7
Wang et al.	2019			★	★			★	3
Zambrano et al.	2018				★★				2
