# Supplementary figures and images for: Pulmonary hypertension and the role of MRI flow assessment: a systematic review
Source: Br J Radiol. 2025 Jul 25;98(1175):1938–46. doi: 10.1093/bjr/tqaf182 (PMC12659743; doi:10.1093/bjr/tqaf182)

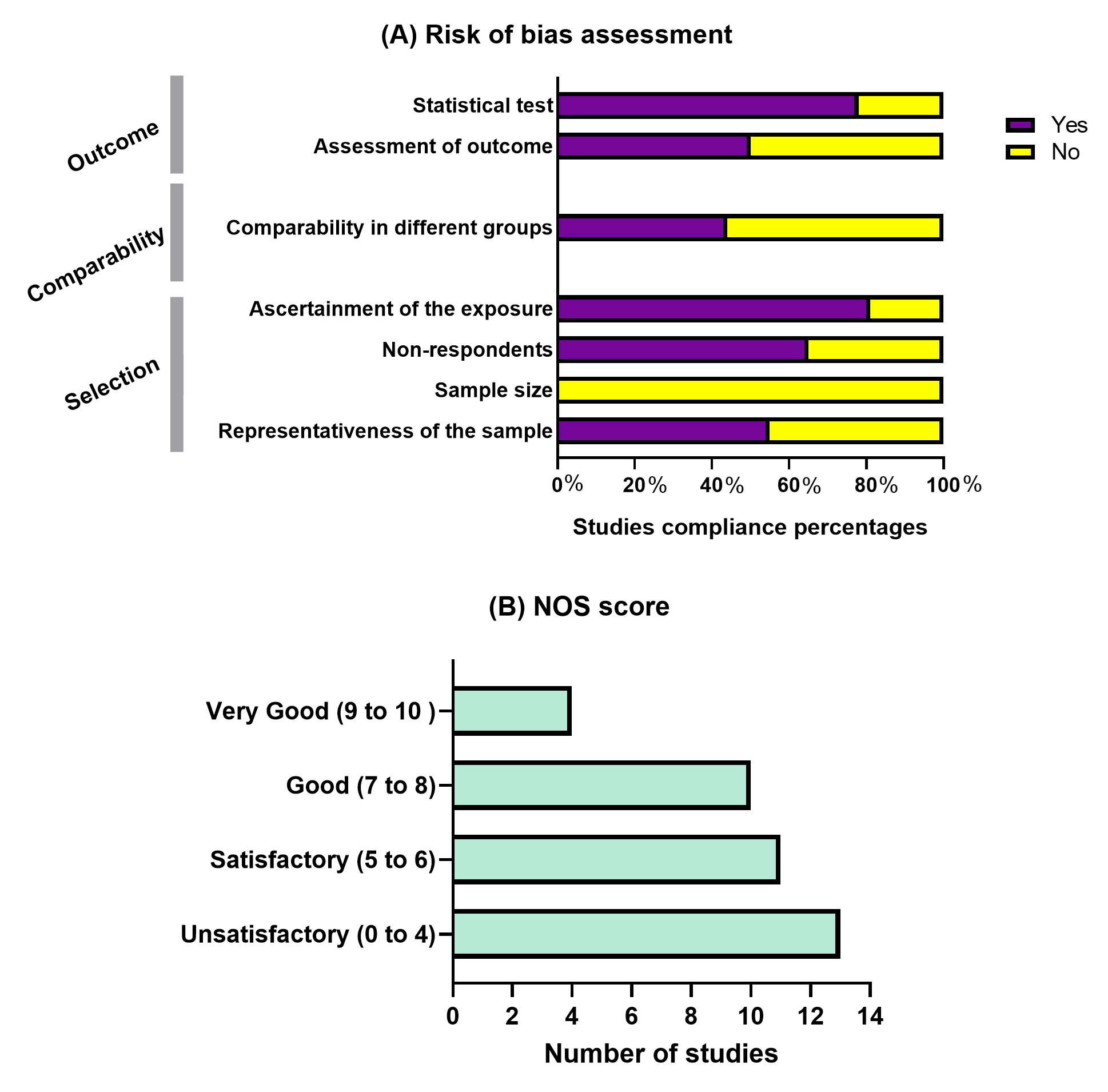

Supplement: tqaf182_Supplementary_Data [file tqaf182_supplementary_data.zip › ROB.png]
